# Memorable meals: The memory-experience gap in day-to-day experiences

**DOI:** 10.1371/journal.pone.0249190

**Published:** 2021-03-30

**Authors:** Karoline Villinger, Deborah R. Wahl, Harald T. Schupp, Britta Renner

**Affiliations:** 1 Department of Psychology, Psychological Assessment & Health Psychology, University of Konstanz, Konstanz, Germany; 2 Department of Psychology, General Psychology, University of Konstanz, Konstanz, Germany; Karl Landsteiner University of Health Sciences, AUSTRIA

## Abstract

Research shows that retrospective memory is often more extreme than in-the-moment experiences. While investigations into this phenomenon have mostly focused on distinct, one-time experiences, we examined it with respect to recurring day-to-day experiences in the eating domain, focusing on variables of the snapshot model—i.e., the most intense and the final experience. We used a smartphone-based Ecological Momentary Assessment to assess the food intake and eating happiness of 103 participants (82.52% female, *M*_age_ = 21.97 years) over eight days, and then calculated their best (positive peak), worst (negative peak) and final experiences. Remembered eating happiness was assessed immediately after the study (immediate recall) and after four weeks (delayed recall). A significant memory-experience gap was revealed at immediate recall (*d* = .53). Remembered eating happiness was predicted by the worst eating experience (β = .41, *p* < .001), but not by the best or final eating experience. Analyzing changes over time did not show a significant memory-experience gap at delayed recall, but did reveal a similar influence of the worst eating experience (β = .39, *p* < .001). Findings indicate that, in the domain of eating, retrospective memory is mainly influenced by negative experiences. Overall, the results indicate that the snapshot model is a valid conceptualization to explain recall of both outstanding and day-to-day experiences.

## Introduction

When we want to remember something from our past such as how much we enjoyed an experience or event, we might ask our friends and family, look at photos, or consult diary entries. Mostly, however, we consult our memories, and these memories of past experiences guide our lives. This can be positive as memories can help us to prepare for future events and act appropriately, but can also be negative as our recollections of negative experiences might be obstacles that discourage us from trying something again. In either scenario, however, we must bear in mind that the experience we remember is not an exact blueprint of what we experienced in the actual moment of the situation. Specifically, while affective feelings are integral for in-the-moment experiences, they are not directly available in retrospective memory and must be reconstructed based on semantic and episodic memory [1].

Much research has been devoted to discrepancies in the details between people’s in-the-moment and retrospective reports of emotional experiences, examining different event types (i.e. positive and negative events), methods of assessing self-reports (i.e. specific and global reports), and approaches to measuring actual experiences (i.e. day- and event reconstruction method and experience sampling [1–4]). One consistent finding from numerous studies exploring different areas and affective events of both positive and negative valence suggests that there is a gap between memory and experience, with retrospective memories tending to be more extreme than in-the-moment experiences [5–9]. Based on extensive studies with hypothetical [10], laboratory [11, 12], experimental [6], and real-life [5] settings, Fredrickson and Kahneman proposed a ‘Snapshot Model’ to explain the discrepancy between in-the-moment ratings and retrospective evaluations [13]. This model suggests that retrospective evaluations are comprised of individual moments [14], with two points in the experience stream having a disproportional influence; the most intense moment (‘peak’) which can be the best or worst experience, depending on its valence [5, 13, 15, 16], and/or the final or last few moments (‘end’) [12, 16]. However, the duration of the experience tends to be neglected, which results in evaluations that are based on a ‘peak-end rule’ [14].

However, as memories and especially event-specific knowledge and memories of affective qualities fade over time [17–20], our evaluations of them start to become more reliant on our general knowledge and beliefs (see also [21, 22]). Robinson and Clore [23, 24] therefore extended the model formulated by Fredrickson and Kahneman [13] to propose an ‘Accessibility Model of Self-Reports’, which takes the finding that specific details of an event or experience will be less accessible over time into account. While it seems that the peak-end rule is a valid explanation for retrospective evaluations after short periods of time, it may not extend to longer retrieval intervals.

Although a remarkable number of studies have demonstrated the presence of a memory-experience gap, it must be noted that they mostly focused on relatively outstanding, distinct, one-time experiences such as medical procedures/pain and vacations, which are not part of normal day-to-day routines [5, 6, 8, 12, 25]. However, life contains many day-to-day experiences that might be mundane rather than outstanding, such as social interactions or eating experiences. While these experiences might not have meaningful impacts by themselves, taken together they define large parts of our daily life. This raises the question of whether memory-experience gaps are also present in familiar day-to-day experiences.

There has not yet been a systematic investigation of bias in the remembrance of day-to-day experiences, of which eating, including multiple eating occasions over the course of a day [26], is one of the most common [27, 28]. Although some studies have already investigated the memory-experience gap in this area, results are mixed regarding the influence of distinct in-the-moment characteristics [29–31]. Furthermore, these studies focused on comparably distinct and short-term laboratory paradigms such as using imagined meals or eating different selected snack foods or single food items, which neglects the great diversity and complexity of eating and the food choices we make in our daily lives.

### The present study

The present study used an event-based Ecological Momentary Assessment (EMA, [32–40] to investigate the memory-experience gap in recurring day-to-day experiences in a real-life ecological setting. The participants were asked to report their food intake and rate their eating happiness (i.e. their experience of pleasure, satisfaction, and taste) for all eating occasions over a period of eight consecutive days. Average retrospective eating happiness for the study period was assessed immediately at the end of the study (immediate recall) and at a follow-up assessment four weeks later (delayed recall). Three hypotheses based on theory and previous findings were tested: Firstly, that memories of eating happiness are expected to overestimate the in-the-moment experience, resulting in a memory-experience gap. Secondly, following the snapshot model, we tested the influence of peak and final experience on immediate recall. As eating itself has no clear positive or negative connotation [36], we included both the best (positive peak) and worst (negative peak) experience as peak experiences. Thirdly, we assessed changes in the influence of immediate and delayed recall to test for the changes across time that the accessibility model of self-reports assumes and examine the temporal gradient of the predictive influence.

## Material and methods

The present study was part of the SMARTACT research project (www.uni-konstanz.de/smartact). It was conducted in accordance with the guidelines of the German Psychological Society (Deutsche Gesellschaft für Psychologie; see http://www.dgps.de/index.php?id=96422; see paragraph C.III) and the Declaration of Helsinki. The study protocol was approved by the University of Konstanz’s Institutional Review Board (Approval number 09/2019) and conformed to ethical guidelines and regulations. All participants gave written informed consent prior to participation. While the present publication focused on in-the-moment experienced and remembered eating happiness, logging behavior and reasons for missing events are reported in Ziesemer et al. ([26]; Study 3), and further data is presented elsewhere [41].

### Participants

The study aimed for a sample size to detect small to moderate effects as no prior information about expected effect sizes of memory-experience gap in the area of everyday eating was available and effects from related research in other domains varied greatly. Therefore, a final sample size of *N* = 96 was targeted in accordance with the power analysis (GPower 3.1.9.4) recommendations of α = .05, power = 0.90 and *f* = .15 for a small to moderate effect (see Cohen’s criteria [42]). Accounting for dropouts, 113 participants took part in an initial introductory session. Two withdrew after starting for personal reasons (i.e. temporal constraints), and another eight had to be excluded as they did not provide complete data sets. The remaining 103 participants (85 females, 82.52%) were mostly students (*n* = 100, 97.1%) from the University of Konstanz with an average age of 21.97 years (*SD* = 5.35, range 18–51 years) and an average BMI of 21.66 kg/m^2^ (*SD* = 2.65, range 16.69–30.12 kg/m^2^). The participants used a smartphone to assess their eating behavior as close as possible to real time and in their natural environment through an event-based Ecological Momentary Assessment.

### Procedure

The sample was recruited via the online study pool of the University of Konstanz and short notices distributed at the university. In an introductory session, the participants were given a short description of the procedure and completed a baseline questionnaire on their smartphones which included descriptive and self-reported anthropometric measures. The participants could choose to use their own smartphone for the assessment where it was feasible (*n* = 49). Alternatively, a smartphone (type Samsung Galaxy J5 (Android 6.0.1) or ASUS Padfone Infinity (Android 5.0.2)) was provided for the duration of the assessment period (*n* = 54). Once the application (app) movisensXS (movisens GmbH Karlsruhe; version 1.1.1; available on Google Play for Android smartphones) had been installed, the questionnaires were downloaded onto the phone. Data was collected between November and December 2017 and was sent to the server automatically via an internet connection. To support data collection and ensure high compliance, the participants were asked to choose individually timed reminders in the morning and evening before the study started. The morning reminder encouraged participants to use the app and record all eating occasions during the day, while the evening reminder prompted them to record any meals or snacks they had missed during the day (see also Ziesemer et al. [26]).

**Fig 1:**
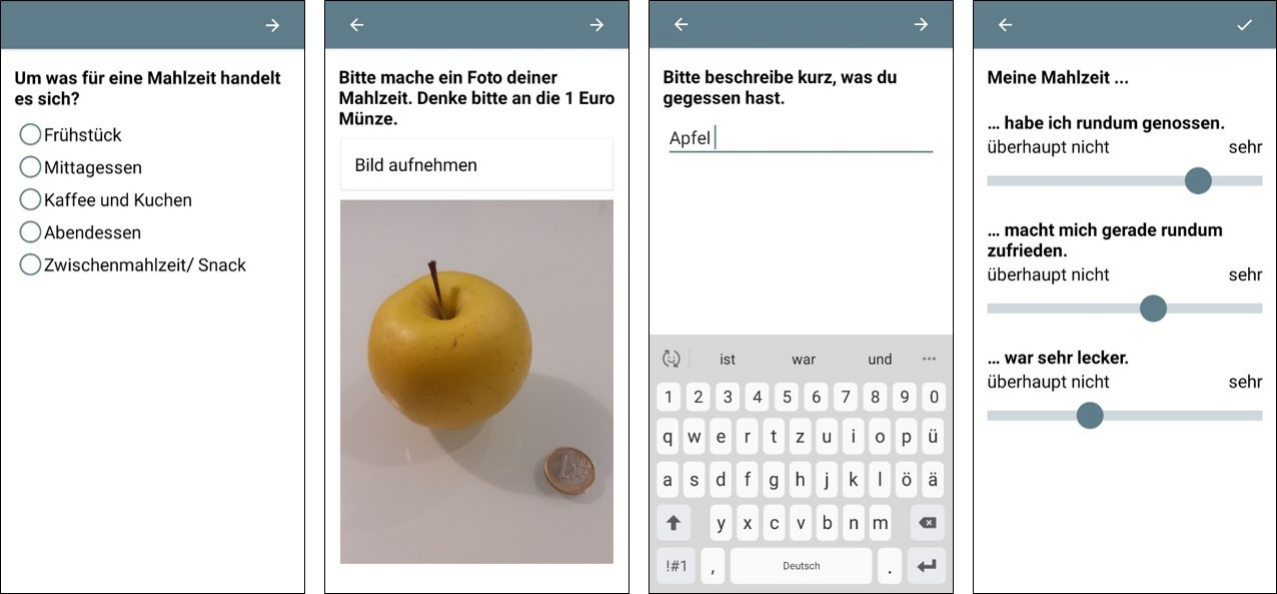
Screenshots of the app for assessing eating events and in-the-moment eating happiness.

During the EMA period, the participants were instructed to access the app to record all eating occasions for eight consecutive days. They were asked to indicate the meal type, take pictures of the meal, describe its main components, and rate their eating happiness (see Fig 1). Meals that they forgot to enter in the moment of consumption could be recorded subsequently; and these recordings were marked as such for the analysis. Fluid intake was not assessed. After recording for eight consecutive days, the participants were sent a questionnaire to retrospectively assess their eating happiness during the study period. Retrospective eating happiness was assessed again after four weeks via an online questionnaire, implemented with the software Qualtrics. For compensation, the participants received either 1½ hours course credits or 15 Euros.

### Measures

Eating happiness of meals was assessed via three items (see also Villinger et al. [36], Wahl, Villinger et al. [37]). The participants were asked to rate (a) how much they enjoy their meal, (b) how pleased they are with their meal, and (c) how tasty their meal is. Ratings were given on a visual slider ranging from ‘not at all’ [0] to ‘a lot’ [100], which had to be activated by the participants clicking on the respective position of the slider. The participants were asked to rate their general eating happiness at the beginning of the study (baseline eating happiness), record their eating happiness every time they entered a meal or snack (in-the-moment eating happiness), and to remember their overall eating happiness as experienced during the study period immediately after the end of the study (immediate recall) and at a follow-up four weeks later (delayed recall). The same three eating happiness items were used for each assessment, although the verb tense changed accordingly. An Intraclass-Correlation-Coefficient (ICC) of .34 was observed for the EMA period, indicating a substantial variation in experienced eating happiness within participants, or between different eating occasions.

### Analytical procedure

Principal axis factor analysis of the three eating happiness items revealed a one factor solution, eigenvalue = 2.48 accounting for 82.68% of the variance (all factor loadings ≥ .90). Therefore, an average score of the three items was used for the analysis. A one-way repeated-measures ANOVA was performed to compare mean level differences between eating happiness experienced in the moment of consumption, at immediate recall and at delayed recall. In addition, the potential influence of variables such as general eating happiness, age, gender, and number of meals reported during the EMA period was analyzed. Since Mauchly’s test indicated that the assumption of sphericity had been violated, *χ^2^*(2) = 0.924, *p* = .018, the Greenhouse-Geisser corrected tests are reported (*ε* = .93). Memory-experience gaps were calculated using the difference between retrospective and in-the-moment eating happiness and further examined using a one-sample t-test. V-plots were created to visualize and compare data distributions for in-the-moment experiences, immediate, and delayed recall (see Blumenschein et al. [43] for a detailed description of the v-plots). Variables were created on the meal level for in-the-moment characteristics and aggregated per day for best (positive peak), worst (negative peak), and final eating occasion or day, respectively. Ratings aggregated per day showed a particularly high correlation with the respective characteristics on the meal level (.71 ≤ *r* ≤ .79, see Table 1), and analyses using aggregated values per day revealed the same pattern of results. Thus, for brevity, only analyses on the meal level are reported in detail. A two-step multiple regression model controlling for baseline eating happiness in the initial step was performed to examine the influence of in-the-moment characteristics on retrospective eating happiness. The relative importance of each predictor was calculated using the R package ‘relaimpo’ [44] to quantify the contribution of each predictor to the multiple regression model. As recommended by Lindeman, Merenda, and Gold [45], the ‘averaging over orderings’ method was used to assess relative importance. All analyses were conducted with IBM SPSS Statistics (version 25 for Windows) and R (version 4.0.3 for Windows).

**Table 1:** Descriptive statistics and correlations of characteristics of in-the-moment experienced eating happiness on the meal level and aggregated per day.

	*M*	*SD*	Range	1	2	3	4	5
**Meal level**								
1. Best eating occasion (pos. peak)	97.45	4.71	73.33 − 100.00					
2. Worst eating occasion (neg. peak)	50.27	16.74	0.00 − 97.67	.17				
3. Final eating occasion	74.88	16.30	40.67 − 100.00	.29**	.33**			
**Aggregated per day**								
4. Best day (pos. peak)	90.47	7.89	61.22 − 100.00	.75**	.33**	.46***		
5. Worst day (neg. peak)	66.36	14.49	19.67 − 99.42	.37**	.79**	.31**	.50**	
6. Final day	79.84	10.91	52.17 − 99.42	.47**	.39**	.71***	.58**	.44**

*Note*. M and SD are used to represent mean and standard deviation, respectively.

** indicates *p* < .01.

## Results

### Descriptive analysis of in-the-moment characteristics

The participants reported a total of 2733 eating occasions over the eight-day mobile assessment period, of which 24.8% were classified by the participants as breakfast, 20.0% as lunch, 24.4% as dinner, 27.7% as snacks, and 3.1% as afternoon tea. They recorded an average of *M* = 27.11 (*SD* = 8.24) meals, ranging from nine to 53 meals across the study period.

Ratings of individual eating occasions revealed pronounced differences between the participants, with ratings for the best (positive peak) ranging from 73.33 to 100.00 (*M* = 97.45, *SD* = 4.71) and for the worst (negative peak) from 0.00 to 97.67 (*M* = 50.27, *SD* = 16.74). There was a notable overlap between the best and the worst eating occasions across participants. Eating happiness ratings for the final eating occasion also showed substantial interindividual variability, with experiences of the final eating occasion ranging from 40.67 to 100.00 (*M* = 74.88, *SD* = 16.30) across the participants (see Table 1).

### Discrepancy between in-the-moment and retrospective eating happiness: The memory-experience gap

**Fig 2:**
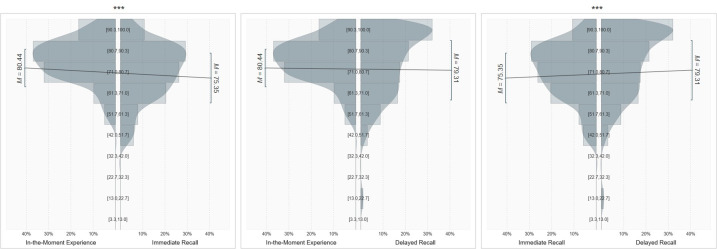
Distribution of eating happiness experienced in-the-moment (across 8 days) and at immediate (immediately after EMA) and delayed (after 4 weeks) recall. *Note*. Smoothed density distributions show the type of distribution and histograms depict the relative frequency of each response category, while means and standard deviations are depicted as lines above the distribution. For comparison, mean values are connected via a line; *** indicates *p* ≤ .001.

Comparing ratings assessed in-the-moment, immediately after the study (immediate recall), and at follow-up (delayed recall) revealed a significant difference between happiness ratings (*F*(1.86, 189.52) = 14.42, *p* < .001, *d* = .53, see Fig 2). As predicted, follow-up testing showed a memory bias with the participants’ remembered eating happiness immediately after the study (immediate recall) (*M* = 75.35, *SD* = 12.68) being significantly lower than their experience in the moment of consumption (*M* = 80.44, *SD* = 9.55), *p* < .001, *d* = .72.

Furthermore, analyzing potential changes over time indicated that retrospective eating happiness after four weeks (delayed recall) (*M* = 79.31, *SD* = 15.16) was significantly higher than retrospective eating happiness immediately after the study (immediate recall), *p* < .001, *d* = .67. However, eating happiness at delayed recall showed no significant difference to eating happiness experienced in-the-moment, *p* = .932 (see Fig 2).

The impact of general eating happiness, age, gender, and number of reported eating occasions during the EMA period were examined to account for potential influences. Regression results showed that general eating happiness assessed at the beginning of the study had a small but significant influence on the magnitude of the memory-experience gap immediately after the study, *F*(1,101) = 10.84, *p* = .001, adjusted *R^2^* = .09, *f^2^* = .10, which was slightly larger for people with higher ratings of general eating happiness at the beginning of the study (β = .31, *p* = .001). However, including age, gender, and number of recorded eating occasions during the EMA period in the next step did not significantly affected the magnitude of the memory-experience gap immediately after the study, *p* > .587.

Analyzing the influence of the variables over time revealed a stable pattern with a comparable small influence of general eating happiness on the magnitude of the memory-experience gap at delayed recall (*F*(1,101) = 9.07, *p* = .003, adjusted *R^2^* = .07, *f^2^* = .07, β = .32) and no significant influence of either age, gender, or the number of recorded eating occasions, *p* > .479.

#### Distribution of the memory-experience gap

**Fig 3:**
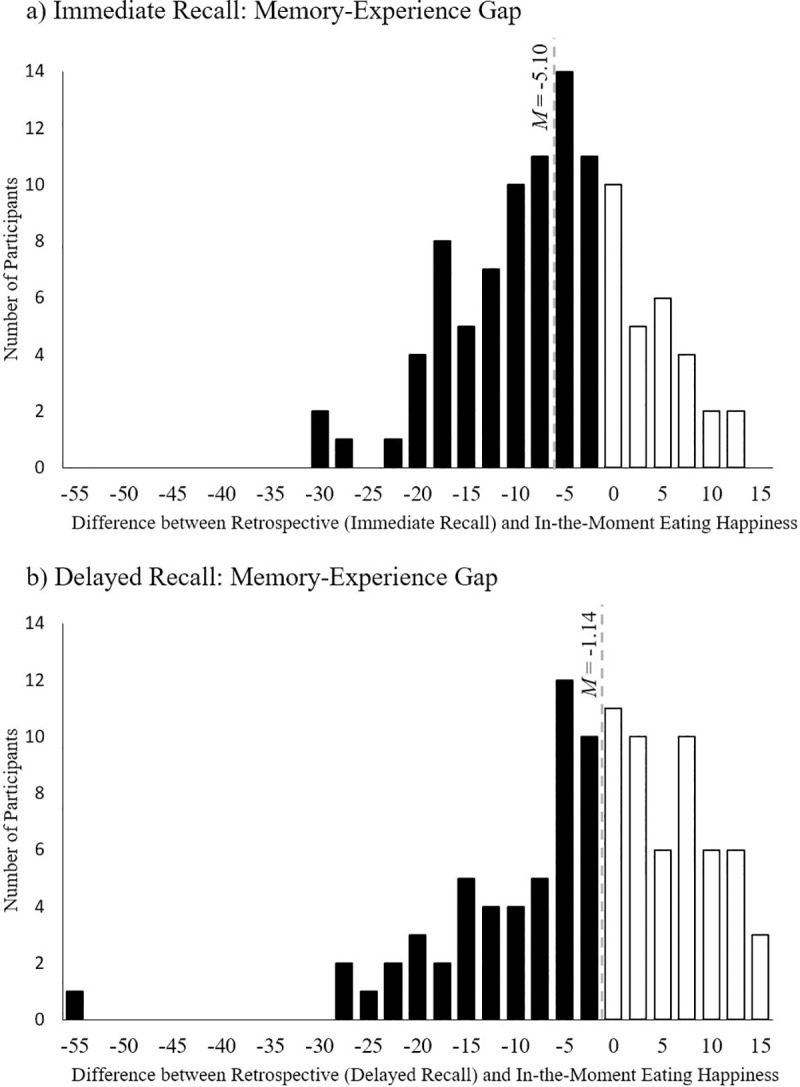
Magnitude and valence of the memory-experience gap for immediate (a) and delayed (b) recall. Bins represent the number of participants.

We also probed the memory-experience gap in more detail regarding the valence (i.e. negative or positive bias) and magnitude of the effect. For immediate recall, most participants (*n* = 74) showed a negative memory bias, while 29 showed a positive memory bias (see Fig 3A). In addition, the bias was more pronounced for participants who showed a negative bias (*M* = -9.14, *SD* = 6.75) than for participants who showed a positive bias, (*M* = 5.23, *SD* = 3.93), *t* (101) = -10.75, *p* < .001.

Analyzing the distribution for delayed recall (see Fig 3B) revealed an equal distribution of participants showing negative (*n* = 51) and positive (*n* = 52) memory bias. However, as with immediate recall, the bias was more pronounced for participants showing a negative bias (*M* = -9.58, *SD* = 9.65) than for participants showing a positive bias, (*M* = 7.15, *SD* = 4.82), *t* (101) = -11.17, *p* < .001.

### Influence of in-the-moment characteristics on retrospective eating happiness: The snapshot model

A two-step multiple regression model was calculated for immediate and delayed recall, controlling for baseline eating happiness in the initial step and entering the best, worst and final eating experience in the second.

For immediate recall, a significant influence was observed for baseline eating happiness, *F*(1,101) = 32.94, *p* < .001, *f^2^* = .32, explaining 24% of the variance in remembered eating happiness immediately after the study. The model was considerably improved by including the distinct in-the-moment characteristics of the experience in a second step, which explained an additional 20% of the variance, *p* < .001. While immediate recall was significantly predicted by the eating occasion with the lowest eating happiness ratings (negative peak, β = .41, *p* < .001), it was not significantly predicted by either the eating occasion with the highest eating happiness (positive peak) or eating happiness experienced at the final eating occasion of the study period (see Table 2). Assessing the relative importance showed that the worst eating occasion had the greatest contribution explaining 49.04% of the variance, followed by baseline eating happiness (31.82%). Overall, baseline eating happiness and distinct in-the-moment characteristics explained 42% of the variance in immediate recall, which can be classified as a large effect, *F*(4,98) = 19.69, *p* < .001, *f^2^* = .72.

**Table 2:** Regression results using immediate and delayed recall as the criterion. Analyses aggregated per day revealed a similar pattern of results as analysis on the meal level.

	Immediate Recall				Delayed Recall			
Predictor	β	β	Corr. *R^2^*	Δ *R^2^*	β	β	Corr. *R^2^*	Δ *R^2^*
		95% *CI*				95% *CI*		
**Meal level**								
**Step 1**			.24				.19	
Baseline eating happiness	.50***	.34 − .67			.45***	.30 − .62		
**Step 2**			.42	.20***			.39	.21***
Baseline eating happiness	.29**	.12 − .46			.23*	.06 − .40		
Best eating occasion	.08	-.08 − .24			.13	-.04 − .29		
(pos. peak)								
Worst eating occasion	.41***	.25 − .58			.39***	.22 − .56		
(neg. peak)								
Final eating occasion	.12	-.05 − .28			.16	-.02 − .33		
**Aggregated per day**								
**Step 1**			.24				.19	
Baseline eating happiness	.50***	.33 − .67			.45***	.27 − .62		
**Step 2**			.46	.24***			.47	.29***
Baseline eating happiness	.28**	.12 − .44			.19*	.04 − .35		
Best day (pos. peak)	.06	-.13 − .25			.13	-.06 − .31		
Worst day (neg. peak)	.47***	.30 − .65			.48***	.31 − .65		
Final day	.06	-.12 − .25			.08	-.10 − .26		

*Note*. β indicates the standardized regression weight. *CI* represents the confidence interval and Δ the change in *R^2^*.

*** *p* < .001

** *p* < .01

* *p* < .05.

While no mean level differences occurred for in-the-moment and delayed recall, the predictive influence of distinct in-the-moment characteristics were analyzed to reveal potential changes over time. As with immediate recall, baseline eating happiness had a significant influence on delayed recall (*F*(1, 101) = 25.12, *p* < .001, *f^2^* = .23), explaining a slightly lower 19% of the variance compared to immediate recall. The additional amount explained by including in-the-moment characteristics in a second step was comparable between immediate and delayed recall. Including in-the-moment characteristics explained an additional 21% of the variance in delayed recall, *p* < .001. Delayed recall was significantly predicted by the eating occasion with the lowest eating happiness ratings (negative peak, β = .39, *p* < .001). Furthermore, as with immediate recall, neither the eating occasion with the highest eating happiness (positive peak) nor the final eating occasion of the study period had a significant impact on retrospective eating happiness after four weeks (see Table 2). Again, assessing the relative contribution of each predictor showed that the worst eating occasion had the greatest contribution (47.05%), followed by baseline eating happiness (25.79%). Overall, the model explained 39% of the variance in delayed recall, which can be classified as a large effect, *F*(4,98) = 17.02, *p* < .001, *f^2^* = .64.

## Discussion

The present study examined eating happiness as an example of a familiar day-to-day experience in real-life. The results displayed a significant discrepancy between in-the-moment and remembered eating happiness immediately after the study, demonstrating a memory-experience gap. While remembered eating happiness was predicted by the eating occasion with the worst eating experience (negative peak), retrospective evaluations were not significantly shaped by the eating occasion with the best eating experience (positive peak) or the experience of the final eating occasion. Analyzing changes over time showed that whilst there was no significant mean level difference at delayed recall, remembered eating happiness was similarly predicted by eating happiness from the worst eating occasion of the study period.

Overall, the participants remembered their eating experiences as being less enjoyable than they felt in the moment of consumption. This finding of retrospective evaluations being lower than actual experiences contradicts the classical pattern found in earlier studies which examined medical procedures/pain or vacation experiences [8, 12, 25]. It does, however, echo findings within the area of eating experiences [30, 46]. It must be noted that eating experiences are qualitatively different to experiences of medical procedures or vacations. While medical or vacation experiences have clear positive or negative connotations, eating experiences are characterized by great inter- and intraindividual variability. Eating experiences, both generally and in relation to the consumption of specific dishes, can be of both negative and positive valence, and both the valence and the intensity can also vary between different eating occasions. This substantial variation and diversity in eating experiences can be seen in the present data in the ICC of .34, which demonstrates a substantial variation of experienced eating happiness across eating occasions within participants. It would be informative to examine the memory-experience gap in more detail to further evaluate the diverse nature of eating experiences, also taking the valence of the gap and potential differential effects into account.

While a continuous and profound assessment of the experience is needed to capture this diversity and complexity, most previous studies have examined rather distinct and short-term eating experiences which focused mainly on the consumption of selected and specific food items [29–31, 47]. This might only provide a very limited and potentially biased picture of the experience, and does not allow generalizations to other foods. It is therefore important to highlight that the results of the present study are based on real-life eating experiences, and so reflect the diversity and complexity of eating and food choices as they occur in daily life, covering both routine and special or novel eating experiences (see Villinger et al. [36] for similar results in forecasted eating happiness). As with previous research, the present study shows that the memory-experience gap is a general phenomenon, which applies to both outstanding/defined and day-to-day life experiences. Future study should explore the extent to which the composition and familiarity of a meal, along with other situational context variables, affects the memorability of specific meals beyond the eating-related hedonic experience.

In line with the snapshot model, we found that peak experiences had a significant influence on remembered eating happiness. However, it is remarkable that we only found a significant influence on retrospective evaluations for negative and not for positive peak experiences (see for similar results Robinson [30]). It has been argued that peak experiences must be seen and experienced as unique to affect retrospective evaluations [48]. Since experienced eating happiness in the moment of consumption was rather high across eating occasions and participants (*M* = 80.44, *SD* = 9.55), experienced positive peaks (*M* = 97.45, *SD* = 4.71) were rather modest compared to the average experience. It might be that the participants did not view the positive peak experiences as distinct and unique, which would explain the lack of a significant influence on retrospective evaluations. Furthermore, Fredrickson [49] raised the assumption that the disproportional influence of distinct in-the-moment characteristics is based on both perceptual salience and personal meaning. Regarding the worst experience, it might be that this observation is specific to the area of eating where bad and possibly harmful eating experiences could threaten both survival and well-being and therefore might be of special relevance from both a personal and evolutionary perspective.

It is also notable that, although it is pervasive in memory research, we did not find final experiences to have a significant influence on retrospective evaluations. To date, most studies have actively manipulated final experiences to examine their influence on recall [12, 29]. In contrast, the present study examined experiences as they occur naturally in real-life without manipulating or stressing any particular experiences such as final ones. Also, rather than being time-bound, assessments of experience in the present study clearly capture chapters within a flow of experience, of which final eating experiences are merely the last ones to be assessed rather than markers for the completion of an experience. Since the aim was to assess real-life eating happiness, and there is no reason to assume that the participants changed their eating behavior after the study ended, it is unlikely that they would have placed any particular importance on their final experiences. Similarly, as argued in relation to positive peaks, final experiences might not have seemed or been experienced as outstanding or of particular importance, which might explain why final experiences had no particular influence on retrospective evaluations [30, 48].

In addition to immediate recall, we also assessed potential changes over time, including delayed recall four weeks after the study period had ended. While most studies also demonstrate memory-experience gaps after considerable time delays of multiple weeks between having and recalling an experience [8, 25], the present data did not show a mean level difference at delayed recall. However, the fact that we focused on memories of real-life day-to-day experiences must be taken into account, and the participants had most likely encountered at least a hundred additional eating occasions during the four weeks between the end of the study and the delayed recall. These newer experiences could have interfered with older memories, especially as these experiences were most likely highly similar to the experiences that were assessed during the study period [50, 51]. While memory interference might explain why we did not find a memory-experience gap at delayed recall, the detailed analysis of distinct in-the-moment characteristics revealed a comparable impact of the worst eating experience (negative peak) on retrospective eating happiness at immediate and delayed recall (β = .41 and β = .39 respectively). This pattern was unexpected, as memories of details are assumed to decrease over time [17, 18]. However, as previously discussed, worst experiences might be of particular importance in the area of eating, explaining their stable impact over time. Nevertheless, it would be informative to examine the temporal pattern and potential qualitative changes in memory for day-to-day experiences in more detail, including longer time periods, and also to make a detailed assessment of the type of information used to generate retrospective evaluations over time (see as example Geng et al. [52]).

## Conclusion

The present study adds to existing literature by stressing the importance of capturing experiences in real-life and in the moment that they occur (see also Schwarz [3] and Villinger et al. [36]). The results contribute to the generalizability of memory biases, demonstrating that biased memory is a general phenomenon that is present in memories of both outstanding events and reoccurring day-to-day experiences. Furthermore, the snapshot model also seems to be a valid conceptualization to explain our recall of day-to-day experiences that are neither distinct nor clearly time bound. However, the data emphasizes that the disproportional influence of distinct moments during an experience on how we remember it is due to the outstanding characteristics of those moments rather than being a general rule.
